# Supplementary material for: Seasonal Influence of Biodiversity on Soil Respiration in a Temperate Forest
Source: Plants (Basel). 2022 Dec 5;11(23):3391. doi: 10.3390/plants11233391 (PMC9738006; doi:10.3390/plants11233391)
Supplement: Supplementary file 1 [file plants-11-03391-s001.zip › plants-2040653-supplementary.pdf]

Table S1. Principal component analysis (loadings) of the soil nutrients across 120 forest plots.

Soil variables	PCA 1 (51.3%)	PCA 2 (18.6%)
pH	-0.18	0.46
Organic matter (g kg^{-1})	0.53	-0.05
Extractable N (mg kg^{-1})	0.29	0.48
Extractable P (mg kg^{-1})	0.10	0.29
Extractable K (mg kg^{-1})	-0.08	0.62
Total N (g kg^{-1})	0.55	-0.01
Total P (g kg^{-1})	0.45	-0.16
Total K (g kg^{-1})	-0.29	-0.24

Table S2. Correlations between plant community structural parameters (Total basal area, Tree $_{BA}$; Tree species richness, Tree $_{SR}$; Tree Shannon–Wiener index, Tree $_{SWI}$) and soil respiration within various radius distances of 5 m, 10 m and 15 m from each measurement point in each season.

Distance	Parameters	Spring	Summer	Autumn
5 m	Tree $_{BA}$	-0.015	0.006	-0.004
	Tree $_{SR}$	0.035	0.064	0.061
	Tree $_{SWI}$	0.028	0.056	0.088
10 m	Tree $_{BA}$	**0.225***	**0.207***	0.138
	Tree $_{SR}$	**0.184***	**0.189***	**0.183***
	Tree $_{SWI}$	0.075	0.112	**0.181***
15 m	Tree $_{BA}$	0.148	0.027	-0.054
	Tree $_{SR}$	0.134	0.124	0.087
	Tree $_{SWI}$	-0.004	-0.017	-0.001

Table S3. spatial autocorrelation analysis of soil respiration

	spring	summer	autumn
Moran I	0.23	0.027	0.016
P value	0.00	0.52	0.67
result	clustered	random	random

The Global Moran's I inflected represents spatial autocorrelation effect strength. P value less than 0.05 indicates the existence of spatial autocorrelation.

Table S4 Pearson's correlation coefficients of all abiotic and biotic variables used in this study. *r* >0.6 are shown in bold. ** *P*<0.01, **P*<0.05.

	Soil $_{PC1}$	Soil $_{PC2}$	Tree $_{BA}$	Tree $_{SR}$	Tree $_{SWI}$	Tree $_{TSV}$	Bacteria $_{SWI}$	Fungi $_{SWI}$	Nematode $_{SR}$	Elevation	slope	convexity
Soil $_{PC1}$												
Soil $_{PC2}$	0											
Tree $_{BA}$	-0.4	0.08										
Tree $_{SR}$	0.32**	-0.07	0.01									
Tree $_{SWI}$	0.21*	0.03	0.05	**0.69****								
Tree $_{SV}$	-0.19*	0.01	0.27**	0.16	0.14							
Bacteria $_{SWI}$	-0.30**	-0.02	-0.07	-0.09	0.02	0.04						
Fungi $_{SWI}$	0.05	0.11	-0.12	0.08	0.06	0.06	0.05					
Nematode $_{SR}$	-0.05	-0.03	-0.10	0.12	0.03	-0.01	0.14	0.11				
Elevation	**-0.61****	0.37**	0.19*	-0.50**	-0.30**	0.15	0.14	-0.05	-0.13			
slope	-0.12	-0.06	-0.26**	0.08	0.07	-0.11	0.23*	0.07	0.33**	-0.30**		
convexity	-0.08	-0.16	0.01	-0.05	-0.11	0.01	0.11	-0.13	-0.07	0.18	-0.01	

Notes: Soil $_{PCA1}$ and Soil $_{PCA2}$, represented eight indexes of soil total and available elements and pH, respectively (Fig. S1), reflecting soil infertile to fertile gradient; Tree $_{BA}$, the sum of basal area of aboveground plants reflects the total biomass of community; Tree $_{SV}$, tree size variation in the plant community; Tree $_{SR}$, the species richness of plant community; Tree $_{SWI}$, the shannon–Wiener index of plant community; Bacteria $_{SWI}$, the Shannon–Wiener index of bacteria; Fungi $_{SWI}$, the Shannon–Wiener index of Fungi; Nematode $_{SR}$, the species richness of nematode; Elevation, Slope and Convexity, topographic index of subsequents

Table S5 Summary of the multiple linear models for soil respiration. Significant effects ($P < 0.05$) are indicated in bold. Beta (β): mean ± standard errors. All predictor abbreviations are explained in Table S4.

Rs in different seasons (R^2)	Predictor	Beta (β)	P-value
Spring (24%)	Slope	-0.04±0.03	0.09
	Convexity	-0.02±0.02	0.37
	SWC	-0.06±0.03	**0.01**
	ST	0.02±0.03	0.46
	Soil $_{PCA1}$	-0.05±0.02	0.05
	Soil $_{PCA2}$	-0.05±0.02	0.05
	Tree $_{BA}$	0.06±0.02	**0.02**
	Tree $_{SR}$	0.09±0.03	**0.00**
	Fungi $_{SWI}$	-0.05±0.02	**0.03**
Summer (51%)	Slope	-0.03±0.02	0.18
	SWC	-0.22±0.02	**0.00**
	ST	0.05±0.02	**0.02**
	Soil $_{PCA1}$	-0.02±0.02	0.45
	Soil $_{PCA2}$	-0.04±0.02	0.06
	Tree $_{SR}$	0.04±0.02	0.09
	Fungi $_{SWI}$	-0.02±0.02	0.28
	Bac. $_{SWI}$	-0.02±0.02	0.39
	Nem. $_{SWI}$	-0.01±0.02	0.50
Autumn (20%)	Slope	-0.05±0.03	0.11
	Convexity	0.05±0.03	0.11
	SWC	-0.11±0.03	**0.00**
	ST	0.06±0.03	0.06
	Soil $_{PCA1}$	-0.09±0.03	**0.01**
	Tree $_{BA}$	0.03±0.03	0.28

Tree $_{SR}$	0.09±0.03	**0.00**
Fungi $_{SWI}$	-0.04±0.03	0.14

Table S6 The direct, indirect, and total standardized effects of abiotic and biotic factors in SEM. Models are presented in Fig. 3. Significant effects ($P < 0.05$) are indicated in bold.

**Predictors**	**Pathway to Rs**	**Spring**		**Summer**		**Autumn**	
		Effect	P-value	Effect	P-value	Effect	P-value
Soil nutrients (PCA1)	Direct effect	-0.155	0.075	-0.070	0.322	-0.221	**0.014**
	Indirect effect via Soil water content	-0.039	0.102	-0.089	0.126	-0.035	0.171
	Indirect effect via Tree biomass	-0.006	0.769	0.000	0.986	-0.002	0.803
	Indirect effect via Stand structure	-0.003	0.825	0.001	0.952	-0.005	0.735
	Indirect effect via Tree diversity	0.115	**0.006**	0.078	**0.013**	0.094	**0.018**
	Indirect effect via Soil biodiversity	-0.006	0.775	-0.002	0.81	-0.008	0.555
	Subtotal indirect effect	0.061	0.287	-0.012	0.861	0.044	0.415
	Total effect	-0.094	0.295	-0.082	0.354	-0.177	**0.045**
Soil water content	Direct effect	-0.182	**0.027**	-0.633	**0.000**	-0.252	**0.003**
	Indirect effect via Tree biomass	-0.011	0.593	0.000	0.98	-0.007	0.518
	Indirect effect via Stand structure	-0.003	0.825	0.001	0.952	-0.005	0.734
	Indirect effect via Tree diversity	0.018	0.533	-0.016	0.402	-0.033	0.175
	Indirect effect via Soil biodiversity	-0.02	0.327	-0.014	0.29	0.009	0.513
	Subtotal indirect effect	-0.016	0.715	-0.03	0.321	-0.037	0.249
	Total effect	-0.197	**0.028**	-0.663	**0.000**	-0.289	**0.001**
Tree biomass (Tree $_{BA}$)	Direct effect	0.210	**0.011**	0.002	0.980	0.067	0.431
	Indirect effect via Stand structure	0.005	0.824	-0.001	0.952	0.007	0.732
	Indirect effect via Soil biodiversity	0.026	0.211	0.009	0.414	0.019	0.261
	Subtotal indirect effect	0.031	0.305	0.008	0.677	0.026	0.339
	Total effect	0.242	**0.003**	0.010	0.888	0.093	0.259
Stand structure (Tree $_{SV}$)	Direct effect	0.019	0.824	-0.004	0.952	0.031	0.730
	Indirect effect via Tree diversity	0.077	**0.026**	0.045	0.058	0.05	0.074

	Subtotal indirect effect	0.077	**0.026**	0.045	0.058	0.05	0.074
	Total effect	0.097	0.277	0.041	0.567	0.081	0.368
Tree diversity (Tree $_{SR}$)	Direct effect	0.327	**0.000**	0.213	**0.003**	0.250	**0.005**
	Indirect effect via Soil biodiversity	-0.013	0.515	-0.008	0.451	-0.007	0.601
	Subtotal indirect effect	-0.013	0.515	-0.008	0.451	-0.007	0.601
	Total effect	0.314	**0.000**	0.205	**0.004**	0.243	**0.007**
Soil biodiversity (Fungi SWI)	Direct effect	-0.204	**0.011**	-0.095	0.149	-0.134	0.104
	Indirect effect	-	-	-	-	-	-
	Total effect	-0.204	**0.011**	-0.095	0.149	-0.134	0.104

Table S7 The relative contribution of soil fungal components

	Richness (%)	Abundance (%)
PF	4.0	7.2
EcM	14.3	40.8
AMF	0.4	0.04
SAF	27.3	24.2

Notes: PF, pathogenic fungi; EcM, ectomycorrhzal fungi; AMF, arbuscular mycorrhizal fungi; SAF, saprophytic fungus.

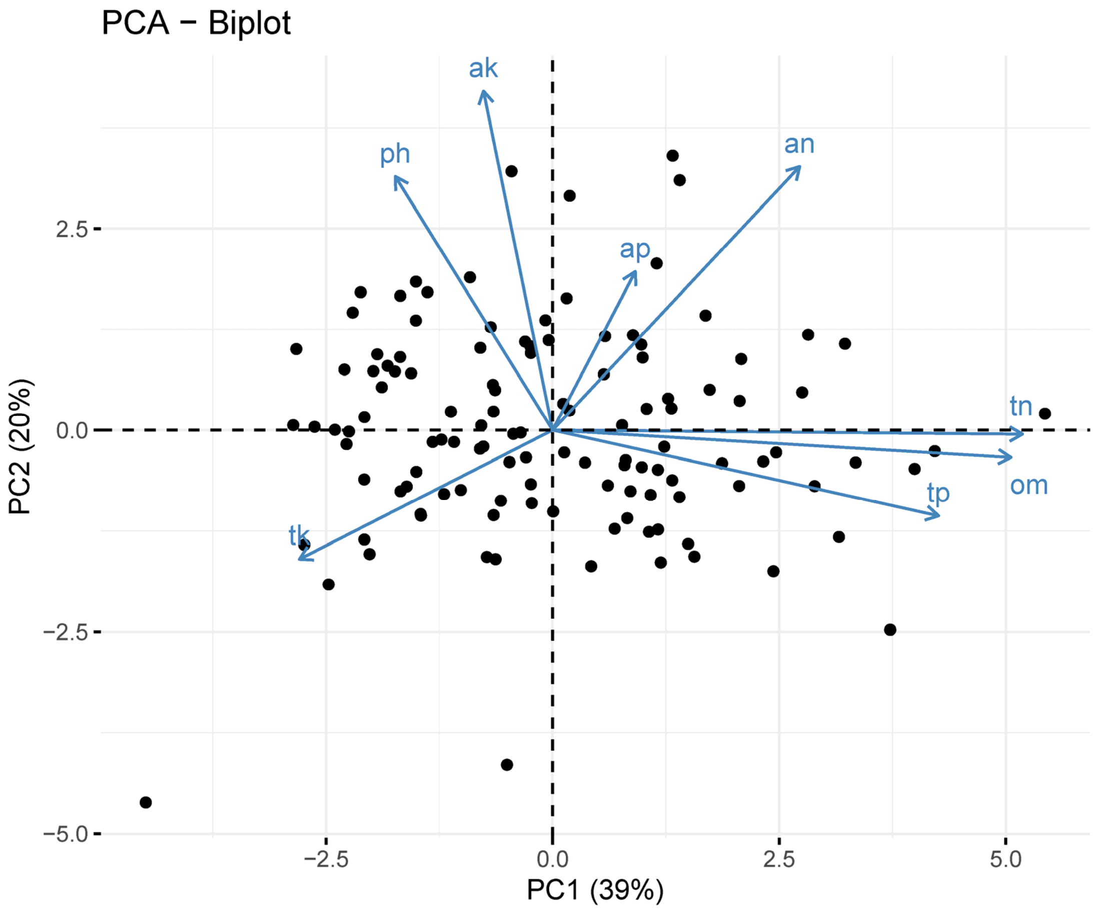

Figure S1. Principal component analysis (loadings) of the soil nutrients across 120 forest plots. (om, organic matter; an, extractable nitrogen; ap, extractable phosphorus; ak, extractable potassium; tn, total nitrogen; tp, total phosphorus; tk, total potassium)

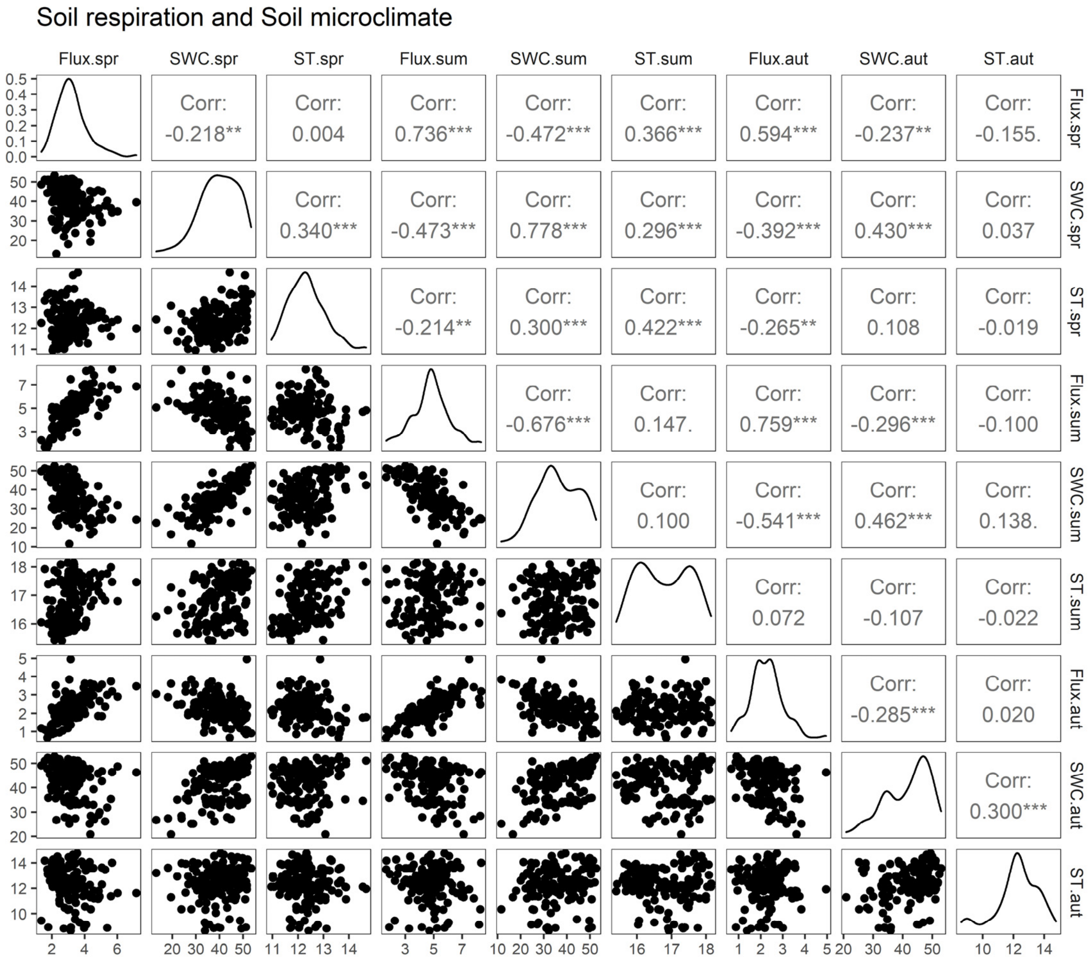

Figure S2 The pairwise correlations of soil respiration and soil microclimate

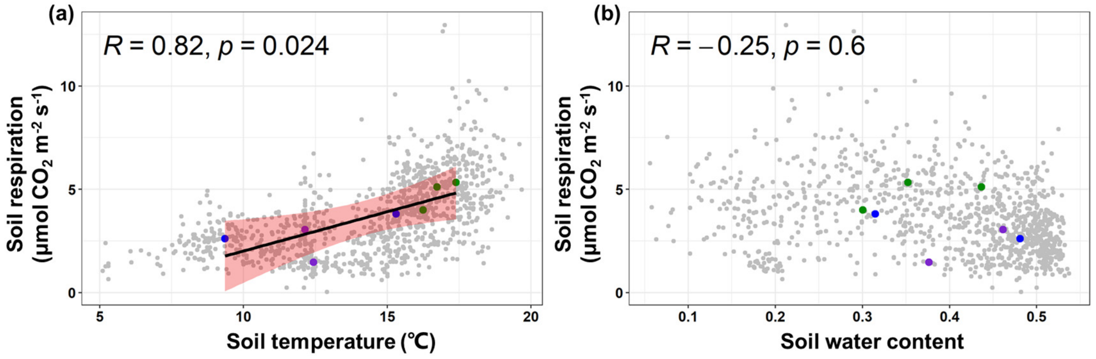

Figure S3 Relationships between temporal variation in mean soil respiration in the 25 ha area across the study period with soil temperature (a) and soil water content (b). Each black point represents the mean value of the 150 sampling points in each measurement campaign. The blue, green and purple points represented measurements in spring, summer and autumn.

(a) **Conceptual model for hypothesized causal paths**

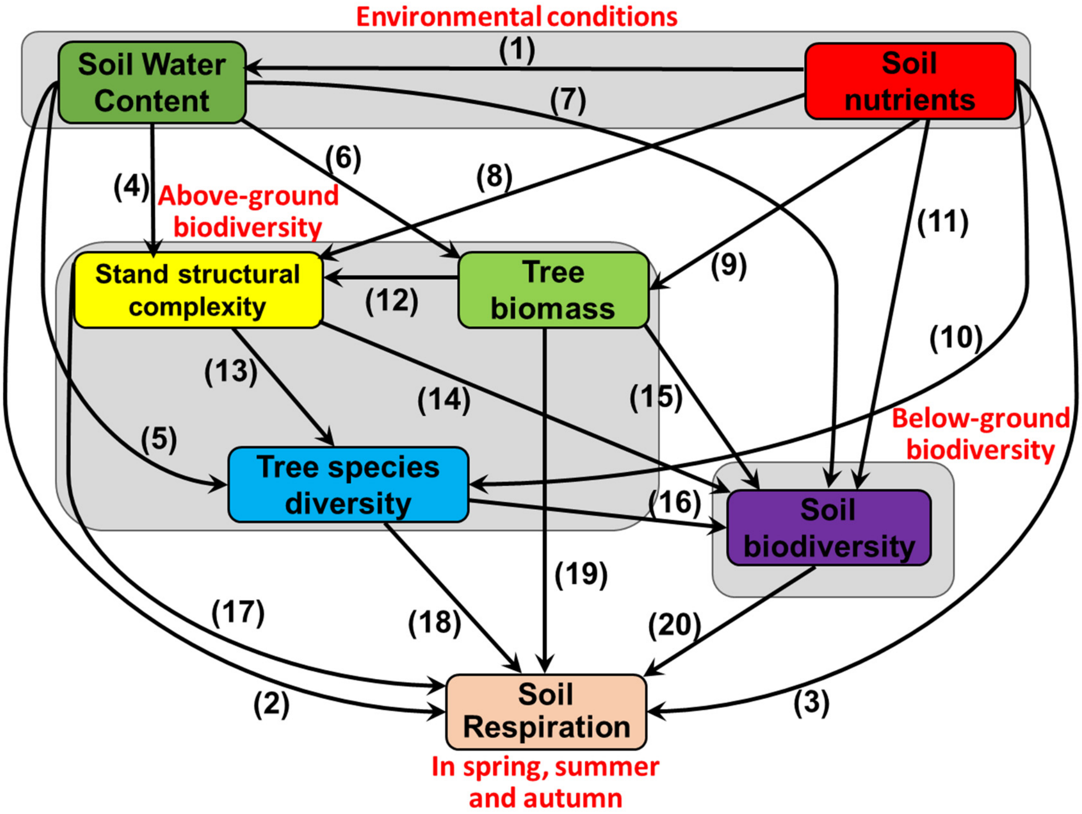

(b) **Schematic illustration of ecological hypothesis/theories/mechanisms/paths**

Pathway No.	Hypothesized pathway	Ecological theories, hypothesis, mechanisms and/ or processes (explanation to each pathway)
(1)	Soil nutrients → Soil water content	Soil nutrients affect soil water content through the relationship with soil water holding capacity
(2)	Soil water content → Soil respiration	Soil water content directly affects soil CO_2 emission by creating anaerobic environment, and indirectly affects autotrophic heterotrophic respiration by affecting plant and microbial activities.
(3)	Soil nutrients → Soil respiration	Fertile soils could directly relieve species from limited resources, leading to high respiration. But high soil fertility could also promote tree competition, with high mortality and turnover rates.
(4)	Soil water content → Stand structure	Soil moisture conditions affect tree growth, thus forming different stand structure complexity.
(5)	Soil water content → Tree species diversity	Soil water availability intensifies interspecific competition and affects plant diversity.
(6)	Soil water content → Tree Biomass	Soil moisture directly mediates photosynthesis and thus affects tree biomass accumulation.
(7)	Soil water content → Soil biodiversity	Soil water content affects soil microbial community structure and diversity through interspecific competition and individual metabolism.
(8)	Soil nutrients → Stand structure	Forests on nutrient-rich soils can develop more structured and dense canopies.
(9)	Soil nutrients → Tree biomass	Promoting tree biomass by providing essential elements such as carbon, nitrogen and phosphorus.
(10)	Soil nutrients → Tree species diversity	Soil resource availability can increase tree diversity because of niche creation, or the competitive advantage of certain species.
(11)	Soil nutrients → Soil biodiversity	Soil organic matter can determine the composition and distribution of soil microbial diversity in natural forests.
(12)	Tree biomass → Stand structure	In natural forests, large trees not only have high biomass but also form complex vertical stand structure due to canopy shading.
(13)	Stand structure → Tree species diversity	The complexity of stand structure determines abiotic factors such as light environment under forest, which affects plant diversity.
(14)	Stand structure → Soil biodiversity	Plant community attributes could affect soil microbial communities by affecting the quantity and quality of litter and root exudates in forest ecosystems.
(15)	Tree biomass → Soil biodiversity	
(16)	Tree species diversity → Soil biodiversity	
(17)	Stand structure → Soil respiration	Stand structure affects understory light environment, thus affecting understory microclimate and soil respiration
(18)	Tree species diversity → Soil respiration	Interspecific competition caused by tree diversity affects plant community metabolic rate and soil autotrophic respiration.
(19)	Tree biomass → Soil respiration	Tree biomass represents the absolute amount of photosynthesis and respiration of plants.
(20)	Soil biodiversity → Soil respiration	Soil biodiversity and community composition regulate soil microbial competition and community respiration rates.

Figure S4 A conceptual model (a) and schematic illustration of ecological hypotheses/theories/mechanisms (b) to test the effects of above- and below-ground community attributes on soil respiration along a local-scale environmental gradient in temperate forests. (a) The hypothesized causal relationships amongst environmental conditions (soil water content and soil nutrients), aboveground community attributes (tree species diversity, tree biomass, stand structural diversity), below-ground biodiversity (i.e. microbes) and soil respiration. The numbering of each hypothesized path is explained in (b) as well as in the introduction. (b) Brief description of ecological hypotheses/theories/mechanisms in relation to hypothesized paths in (a)
